# Annotations

**Published:** 1910-11-12

**Authors:** 


### Annotations.

### Postmen and Lifts.

The Postmaster-General is to be congratulated on the firm stand that he has made, in the interests of his employés, against the claims of tenement-dwellers to have their letters delivered to them by a postman who has to climb several stories in order to get rid of the contents of his bag. The public is generally thoughtless in matters where its own convenience is involved—thoughtless and illogical at the same time. The tenement-dweller argues that because he can do without a lift, the postman can equally well afford to dispense with that luxury. He forgets that the latter has had to tramp several miles in the course of the day, and that there is nothing more wearying to a tired man than to lift his feet a distance of several inches several hundred times in succession. Anyone who has attempted to ascend the Monument twice on the same day will have some conception of the amount of physical exertion and consequent fatigue it involves. From a hygienic point of view no flat or tenement house should be erected without a lift. That, of course, is a matter for the flat-dwellers to agitate about, and if they elect to live several stories from the earth and to indulge in the wearying ascents and descents that such a choice involves they are perfectly free to do so. But it is quite another matter when they maintain that Post Office or any other officials should be subjected to the same strain and the same fatigue in consequence of their exercise of apparent free will. We would like to see the new regulations for postmen extended so as to include telegraph lads and messenger boys as well. When the public finds that living in liftless flats entails more inconveniences than trudging up interminable flights of stairs themselves, it will realise that such dwellings are out of place in our modern civilisation.

### Jews in Medicine.

A few weeks ago, in all innocence, a statement was made in these columns to the effect that medicine as a profession is underpaid, and that the most astute, commercially, of the population, the Jews, avoid it for that reason. Both assertions were made in good faith, and both are, we are persuaded, substantially true. The Editor of the *Jewish World*, however, regards the second one as insulting and erroneous as well. It is clear from his comments that he does not dispute the first one, and that being so it is curious that he should object to the other; for surely it is a compliment to the business intelligence of a people—and was so intended and expressed—to say that they avoid a laborious occupation which is badly paid. Be that as it may, the *Jewish World* would appear to suggest that Jews do not avoid the medical profession. It was not stated that there are none of them at all within our ranks, but only that there are comparatively few; and this is a matter of common knowledge in medical circles. Consider the number of Jewish families enjoying the degree of affluence necessary to the education of a son to medicine; consider, further, the proportion of such sons to those of Christian youths of like circumstances; it cannot be disputed that the percentage of the former entering the profession of medicine is much smaller than of the latter. The *Jewish World* says that we made this a cause of complaint against the Jews; the complaint exists entirely in the editor's imagination. He then branches off into a eulogy of Jewish doctors who work in slum practices, and into denunciations of the apathy of British local government in matters of hygiene. On these points we are in entire agreement, and would desire only to add that for every doctor of the Hebrew people doing his level best to counteract the squalor and disease of large towns, there are hundreds, if not thousands, of Anglo-Saxons.

### Prosecutions in Paddington.

At the beginning of this year attention was called in these columns (The Hospital, January 1, 1910, p. 379) to the fact that the Notification of Births Act of 1907 is being widely neglected and disobeyed by the profession. In Kensington the medical officer of health circularised the doctors in his district with a view to obtaining proper compliance with the Act, and several parents were requested to explain their omission to fulfil the duty which is laid jointly upon them and their medical attendant. In Paddington matters have advanced a step further, for the medical officer of health there has secured two prosecutions; in one case both father and doctor were convicted and mulcted in small fines, in the other the case was dismissed. It is, of course, impossible to judge fairly of a witness's evidence in court from the abbreviated report in a daily paper; but the reporters who attended this trial convey the impression—rightly or wrongly—that the medical officer of health for Paddington (Dr. Dudfield) and some of the local practitioners are not on very friendly terms. The former is reported to have said in effect that the Act is a most valuable one, but is boycotted by the medical profession because no fees attach to its fulfilment. It is regrettable that Dr. Dudfield should say things of this sort, but it is much more regrettable if—as may be supposed—he has solid grounds for his assertion. The medical profession most certainly does regard it as unfair that work should be demanded from it by the State without any recompense, a view which has always been upheld in The Hospital; but now that the Act is law, it ought to be carried out scrupulously. Let efforts be made to amend it by all means, so that some small remuneration shall attach for this service in the cause of public health; but, meanwhile, it should not be possible for accusations such as this to be justly levelled. It will be interesting to see whether other boroughs follow the lead given, and prosecute for omissions under the Act; penalties will no doubt at first remain nominal, and it behoves all who conduct obstetric cases to take the plain hint thus given.

